# A comparison of serotonin neuromodulation of mouse spinal V2a interneurons using perforated patch and whole cell recording techniques

**DOI:** 10.3389/fncel.2012.00039

**Published:** 2012-09-28

**Authors:** Shelby Dietz, Andreas Husch, Ronald M. Harris-Warrick

**Affiliations:** Department of Neurobiology and Behavior, Cornell UniversityIthaca, NY, USA

**Keywords:** Chx10, spinal cord, whole cell recording, locomotion

## Abstract

Whole cell recordings (WCRs) are frequently used to study neuronal properties, but may be problematic when studying neuromodulatory responses, due to dialysis of the cell's cytoplasm. Perforated patch recordings (PPR) avoid cellular dialysis and might reveal additional modulatory effects that are lost during WCR. We have previously used WCR to characterize the responses of the V2a class of Chx10-expressing neurons to serotonin (5-HT) in the neonatal mouse spinal cord (Zhong et al., 2010). Here we directly compare multiple aspects of the responses to 5-HT using WCR and PPR in Chx10-eCFP neurons in spinal cord slices from 2 to 4 day old mice. Cellular properties recorded in PPR and WCR were similar, but high-quality PP recordings could be maintained for significantly longer. Both WCR and PPR cells could respond to 5-HT, and although neurons recorded by PPR showed a significantly greater response to 5-HT in some parameters, the absolute differences between PPR and WCR were small. We conclude that WCR is an acceptable recording method for short-term recordings of neuromodulatory effects, but the less invasive PPR is preferable for detailed analyses and is necessary for stable recordings lasting an hour or more.

## Introduction

Most studies investigating the effects of neuromodulators on vertebrate neurons have used traditional whole cell recordings (WCRs), which break the membrane under the recording pipette and within minutes replace the neuron's cytoplasm with the pipette internal solution (Hamill et al., 1981). Many ionic currents and cellular processes are adversely affected by the washout of intracellular molecules (Becq, 1996; Sarantopoulos, 2007), including second messenger pathway enzymes (Armstrong and Eckert, 1987; Chad et al., 1987), protease inhibitors (Chad and Eckert, 1986; Belles et al., 1988a,b), and calcium buffers (Korn and Weight, 1987; Korn and Horn, 1989). Some rundown can be reduced or prevented by introducing additives to the intracellular pipette solution, and not all currents and processes are sensitive to washout (Sarantopoulos, 2007). Perforated patch recordings (PPR) mitigate intracellular dialysis by using ionophores to open small pores in the membrane, which allow small ions but not large signaling molecules to pass through the membrane (Lindau and Fernandez, 1986; Horn and Marty, 1988; Rae et al., 1991).

Since neuromodulators often act via cytoplasmic second messenger pathways, some effects of neuromodulation may be lost due to the WCR dialysis of the cytoplasm. Despite the advantages offered by protection from intracellular dialysis, the PPR method has not achieved as wide use as traditional WCR in studying neuromodulation, in part because of the increased difficulty of achieving a gigaseal and the long perforation time before recording can begin. Soon after the perforated patch technique was developed, it was shown that cultured mouse brown fat cells recorded with PPR responded to norepinephrine with large changes in membrane conductance, whereas previous WCRs had shown no response to norepinephrine application (Lucero and Pappone, 1989, 1990). Since that time, however, when WCR and PPR have been used side by side in studies of neuromodulation, PPR has typically been applied to a subset of cells to confirm WCR results, and a detailed comparison of multiple properties in WCR and PPR has not been within the scope of the study. The effects of one neuromodulator, dopamine, were reported as no different between PPR and WCR in some cell types (Liu and Lasater, 1994; Gulledge and Jaffe, 1998; Ding and Perkel, 2002) while in others the dopamine effect was larger or qualitatively different in PPR recordings even with only three or four cells recorded in PPR (Aosaki et al., 1998; Ingram et al., 2002). Direct comparisons of the effect of recording type on the action of neuromodulators including serotonin, using large enough sample sizes to allow for thorough analysis, are still needed.

The PPR technique has been shown to be superior to WCR for studies of neuromodulation in some cell types. One class of neurons for which responses to neuromodulation are critical for their functioning is the interneurons (INs) of the locomotor central pattern generator (CPG) in the spinal cord. Genetic analysis has identified the V0, V1, V2a, V2b, and V3 subgroups of spinal INs which are components of the spinal locomotor CPG in the mouse (Kiehn, 2006; Goulding, 2009). The V2a INs are excitatory, ipsilaterally projecting, and express the Chx10 transcription factor (Al-Mosawie et al., 2007; Lundfald et al., 2007). They are components of the locomotor CPG network, and help organize left-right alternation during fictive locomotion (Crone et al., 2008, 2009). In a previous study using WCR (Zhong et al., 2010), we examined the effect on neonatal V2a INs of serotonin (5-HT), a neuromodulator which plays an important role in the control and initiation of locomotion (Jacobs and Fornal, 1993; Schmidt and Jordan, 2000; Hochman, 2001). 5-HT depolarizes many V2a INs and increases their excitability in response to depolarizing current steps (Zhong et al., 2010). Although we do not yet know which 5-HT receptors are expressed in V2a INs, all but one of the known 5-HT receptors are metabotropic; these include the 5-HT_1_ and 5-HT_4−7_ classes acting via the soluble cAMP pathway, which is known to be vulnerable to washout during WCR (Chad and Eckert, 1986; Armstrong and Eckert, 1987; Chad et al., 1987). Considering the possible washout of intracellular messengers during WCR, the responses to 5-HT that we previously recorded may have been weakened or distorted. A recording method that could reveal a stronger or qualitatively different response to neuromodulators in these cells would greatly improve our understanding of the role of neuromodulation in locomotion. We tested the hypothesis that PPR would yield stronger responses to 5-HT by monitoring the effect of WCR and PPR methods on the 5-HT neuromodulation of the V2a INs in the neonatal mouse spinal cord.

## Materials and methods

### *In vitro* slice preparation

V2a cells were fluorescently labeled with eCFP in the Chx10::eCFP line generated by Dr. Steven Crone and Dr. Kamal Sharma at the University of Chicago (Zhong et al., 2010). All preparations were performed in accordance with Cornell University Institutional Animal Care and Use Committee and National Institutes of Health guidelines.

Neonatal (P2–P4) male and female Ch×10::eCFP mice were used in this study. These ages were chosen because virtually all spinal cord slice studies of locomotion-related INs use neonates, and we wished our results to be relevant to these papers. Mice were euthanized by decapitation and their spinal cords removed by laminectomy (Kudo and Yamada, 1987; Jiang et al., 1999) in ice-cold (4°C), oxygenated (95% O_2_/5% CO_2_) glycerol-based artificial cerebrospinal fluid (ACSF) (in mM: 222 glycerol, 3.08 KCl, 1.25 MgSO_4_, 1.18 KH_2_PO_4_, 2.52 CaCl_2_, 25 NaHCO_3_, and 11 D-glucose). The meninges were removed and the upper lumbar cord embedded in agarose. Transverse slices (250 μm) were prepared using a vibrating microtome (HM 650 V, Thermo Scientific). The slices were maintained and recordings made in normal ACSF (in mM: 111 NaCl, 3.08 KCl, 1.25 MgSO_4_, 1.18 KH_2_PO_4_, 2.52 CaCl_2_, 25 NaHCO_3_, and 11 D-glucose) at a flow rate of ~2 ml/min.

### Electrophysiological recordings

Acute slices were visualized using a fixed-stage upright microscope (BX51WI, Olympus) with a 60X water-immersion objective with infrared differential interference contrast optics. eCFP+ INs were identified by epifluorescent illumination.

WCRs were performed as described by Zhong et al. (2010). Briefly, borosilicate glass pipettes with a tip resistance of 3–5 MΩ were filled with an internal solution containing (in mM): 138 K-gluconate, 10 HEPES, 5 ATP-Mg, 0.3 GTP-Li, and 0.0001 CaCl_2_, pH 7.4 with KOH. Pipettes were visually guided to eCFP+ cells and positive pressure was applied through the pipette as it approached the cell. When the pipette pressed against the cell, the positive pressure was quickly released and mouth suction applied until the resistance increased past 100 MΩ. The seals typically reached a gigaohm resistance in less than one minute. Once the resistance reached 1.5 GΩ or higher, a quick pulse of mouth suction was applied to break the membrane under the pipette. A Multiclamp 700 B amplifier and Clampex pClamp 9 software (Molecular Devices, Palo Alto, CA) were used to collect electrophysiological data.

PPR were performed as previously described (Husch et al., 2011). A HEPES-buffered pipette solution was prepared with (in mM) 135 K-gluconate, 10 KCl, 10 HEPES, 0.1 EGTA, and 2 MgCl_2_. Shortly before recording, 1.2 mg Amphotericin-B (Sigma) was added to 20 μl fresh DMSO and vortexed until fully dissolved. 1 mg Pluronic F127 (Sigma) was added to another 40 μl of fresh DMSO and held at 37°C for about fifteen minutes until dissolved. The Amphotericin and pluronic solutions were combined, vortexed, and then added to one ml of filtered pipette solution and vortexed. The prepared Amphotericin solution could be held at room temperature and used for about one hour before losing its efficacy. To record from a cell, a 3–5 MΩ borosilicate glass pipette was tip-filled with filtered pipette solution and back-filled with Amphotericin solution. Working quickly, the pipette was lowered onto an identified Chx10-eCFP cell. Positive pressure was applied for less than one minute on approach to the cell, then switched to negative pressure once the pipette touched the cell membrane to form a gigaseal. Over 30–90 min after the seal was formed, amphotericin diffused to the tip, and formed a low (15–30 MΩ) series access resistance to the interior of the neuron. Cells were visualized and recordings made with the equipment listed above.

All recordings were made in the presence of blockers of fast synaptic transmission: strychnine (10 μM), and picrotoxin (10 μM), and either 2-amino-5-phosphonopentanoic acid (APV, 10 μM) with 6-cyano-7- nitroquinoxaline-2,3-dione disodium salt (CNQX, 10 μM) or kynurenic acid (1 μM).

### Analysis and statistics

All data were analyzed using Excel (Microsoft), Clampfit (Axon Instruments), JMP (SAS), and MATLAB (MathWorks). For action potential (AP) analysis, the membrane potential was adjusted to set the firing frequency at 1 Hz or lower to elicit temporally isolated APs. The voltage threshold for AP generation was measured as the peak of the second derivative of voltage with time during the rising phase of the AP (Zhong et al., 2010). The spike amplitude was measured from the peak of the AP to the trough of the afterhyperpolarization (AHP). The AP half-width was established at the voltage halfway from the spike threshold to the peak of the AP. To assure comparable measures of the membrane input resistance, rheobase, and neuronal excitability across neurons, all neurons were held below threshold at −60 mV with holding current. The input resistance was measured by averaging the voltage response to small hyperpolarizing current pulses from −60 mV. The minimal current injection sufficient for spike generation from −60 mV was defined as the rheobase. For measures of neuronal excitability, a series of depolarizing current steps of increasing amplitude were given, to evoke increased spiking, and a frequency-current (F-I) analysis was performed by measuring the spike frequency as a function of current step amplitude. The instantaneous frequency was defined as the inverse of the interval between first and second APs, while the mean frequency was the average frequency of APs during a one-second current step (Zhong et al., 2010).

Individual criteria were compared by non-parametric Wilcoxon rank sum tests for PPR versus WCR in different cells, and by Wilcoxon signed rank test for control versus 5-HT in the same cell. Fisher's exact test was used to compare 2 × 2 contingency tables. The difference in response to 5-HT was measured using regression analysis, with the absolute difference in a parameter under control conditions and during 5-HT as the dependent variable and recording type and initial value of the parameter in control conditions as independent variables. If the initial value variable was not significant it was removed from the model. To meet the assumptions of the regression model, data with very non-normal distributions were transformed, and in one case an outlier that did not affect the *p*-value was excluded (Neter et al., 1996). F-I regressions used a random coefficient model to correct for multiple measurements from each cell. The dependent variable was the absolute difference in the firing rate between control and 5-HT, while the fixed independent variables were recording type, initial value, and their interactions. Cell identity was entered into the model as a random effect, and non-significant effects were removed from the model. In the box plots, the extent of the box indicates the 25th and 75th percentiles, and the central line represents the median. Whiskers extend to maximum and minimum values within *q*3 + *w*(*q*3 − *q*1) or smaller than *q*1 − *w*(*q*3 − *q*1), where *q*1 and *q*3 are the 25th and 75th percentiles, respectively, and *w* = 1.5. Any values outside these boundaries are outliers and are plotted as a cross. Significant effects were defined as *p* < 0.05. All error bars represent the standard deviation.

## Results

### Baseline intrinsic properties of V2a INs using PPR and WCR

**Figure 1:**
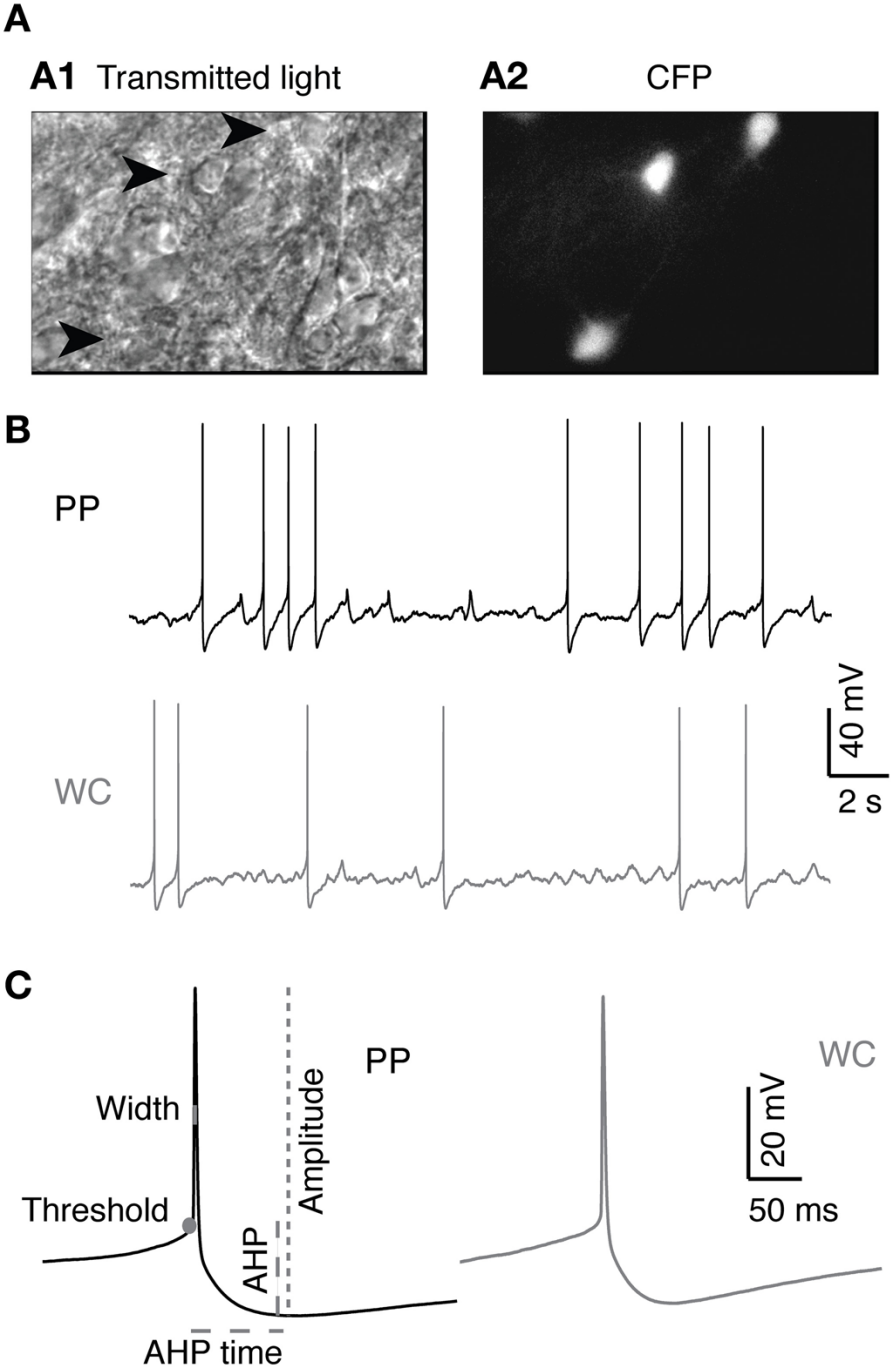
**V2a INs in PP and WC recordings. (A)** Identification of V2a interneurons in a neonatal spinal cord slice. (**A1**) Chx10-eCFP neurons from a P3 mouse spinal cord slice seen under infrared transmitted light. Arrowheads indicate V2a INs. **(A2)** The same region under epifluorescent illumination revealing the CFP-labeled V2a INs. **(B)** Sample traces from PP and WC recordings. **(C)** Action potentials (average of 10 APs) from PP and WC recordings. On the PP action potential, schematics show the measurements of threshold, amplitude, width, afterhyperpolarization (AHP) and afterhyperpolarization time (AHP time), which are described in detail in the “Materials and Methods.”

Before proceeding to test the effect of recording type on the neuromodulatory effects of 5-HT on V2a INs, it was necessary to characterize the baseline intrinsic properties of synaptically isolated V2a INs using our two recording methods. Recordings were made in acute slices of 2–4 day old neonatal mouse spinal cord, in the presence of blockers of fast glutamatergic, GABAergic and glycinergic synaptic activity (Figure 1A). Neurons recorded by the perforated patch method (PP neurons) yielded high quality recordings that lasted far longer than those using the WCR method (WC neurons). WCRs could be maintained for an average of 34 ± 14 min before recording quality deteriorated. Deterioration of the recording was detected principally by slow depolarization, or equivalently an increase in the holding current required for hyperpolarization to −60 mV, and a progressive decrease in AP amplitude (Moyer and Brown, 2002). WC neurons that had deteriorated were typically still able to fire APs and respond to serotonin (5-HT, 10 μM), but showed a combination of reduced access, reduced spike amplitude and increased spike width. Even small reductions in recording quality made it impossible to ensure that washout of 5-HT was complete. PPR, by contrast, could be maintained without decrement for a significantly longer average time of 134 ± 60 minutes (Wilcoxon, *p* = 5.3 × 10^−7^), allowing ample time for complete washout of any drug actions. The longest PPR lasted for four and a half hours, and was voluntarily terminated before any reduction in recording quality was noted. Only 2/20 PP cells deteriorated sufficiently to compromise tests of intrinsic properties and required termination of the experiment, whereas 13/16 WC cells did so (Fisher's exact test, *p* = 0.012). WCR were, however, faster to obtain than PPR.

Most of the intrinsic properties of V2a INs were not significantly different using PPR compared to WCR (Table 1). The same proportion of PP neurons (13/20, 65%) were spontaneously active at rest as WC neurons (7/16, 42%, Fisher's exact test *p* = 0.31), and the mean firing rate and pattern of spontaneous activity of all neurons were not different by recording type (Table 1). When silent cells were excluded, the firing rates of spontaneously firing PP versus WC cells were still not different (Wilcoxon *p* = 0.86). One significant difference between PPR and WCR was the higher input resistance in PPR (Table 1, Wilcoxon *p* = 0.013). Because many neurons were firing too rapidly to define a “resting membrane potential,” we used the holding current required to hyperpolarize the cells to −60 mV (where they were silent) to compare their potentials. The holding current showed a strong trend to be higher in WCR (Table 1, Wilcoxon *p* = 0.0501), which likely results from the lower input resistance in these cells. In addition, we measured the resting potential during the silent periods of cells firing at less than 1 Hz. These weakly firing and silent PP and WC neurons did not have significantly different resting potentials (PPR: −44.4 ± 5.6 mV, WCR: −45.9 ± 6.5 mV; Wilcoxon *p* = 0.40). However, these weakly firing neurons are not necessarily representative of the entire population.

**Table 1:** **Summary baseline intrinsic properties of V2a INs using PPR and WCR**.

**Property**	**Mean PPR (*n* = 20)**	**Mean WCR (*n* = 16)**	***p*-value (Wilcoxon)**
Firing rate (Hz)	0.69 ± 1.3	0.38 ± 0.61	0.31
Input resistance (GΩ)	1.1 ± 0.5	0.8 ± 0.2	0.0130
Holding current (pA)	15.5 ± 10	19.4 ± 7.9	0.0501
AP amplitude (mV)	64.8 ± 6.7	62.6 ± 10.4	0.38
AP threshold (mV)	−30.0 ± 4.2	−27.6 ± 4.6	0.16
AP half-width (ms)	2.3 ± 0.5	2.8 ± 1.0	0.075
AP AHP (mV)	21.8 ± 4.5	17.6 ± 2.9	0.0026
AP AHP time (ms)	54.0 ± 14.5	55.5 ± 17.4	0.96

AP properties were also similar in the two recording conditions (Table 1). The amplitude, threshold, spike half-width, and AHP time of APs were not different for PP versus WC neurons (Table 1; see Figure 1C for illustrations of these parameters). The mean AHP amplitude, however, was significantly more hyperpolarizing using PPR than using WCR (Table 1, Wilcoxon *p* = 0.003). The sag and rebound currents measured in response to hyperpolarizing current steps were also measured, but were not different for PPR versus WCR (data not shown).

### Baseline excitability of V2a INs using PPR and WCR

**Figure 2:**
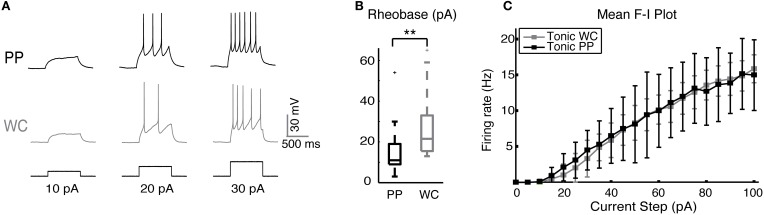
**Excitability of V2a INs in PPR and WCR. (A)** Sample traces from a PP and a WC neuron held at −60 mV, showing responses to injected current steps of 10, 20, and 30 pA. **(B)** The mean rheobase for all PP cells was significantly lower than that of all WC cells. **(C)** F-I plot of the mean frequency of tonic PP (black, *N* = 9) and tonic WC (gray, *N* = 7) cells (instantaneous rate data not shown). The F-I relationship was not different in PP versus WC recordings (regression analysis, mean rate *p* = 0.80, instantaneous rate *p* = 0.81).

We studied the excitability of V2a INs by injecting a series of depolarizing current steps and plotting spike frequencies at each current step (Figure 2A). V2a INs showed similar excitability when recorded using PPR and WCR. The major difference was that the mean rheobase for all PP neurons was slightly though significantly lower than for all WC neurons (Figure 2B, Wilcoxon *p* = 0.002). It is also possible to compare the entire F-I plot for the class of V2a INs that fires tonically with increasing frequency in response to current injections of up to 100 pA (Figure 2C; Zhong et al., 2010). For this subset of tonic cells, the average F-I plot showed no significant difference between PP and WC neurons in either average or instantaneous frequency over all current steps (regression, random coefficient model, mean rate *p* = 0.80, instantaneous rate *p* = 0.81). During a 1-s current step, there was 6% more spike frequency adaptation in PP versus WC neurons (regression, *p* = 0.006; data not shown).

### Effect of serotonin on intrinsic properties of V2a INs using PPR and WCR

**Figure 3:**
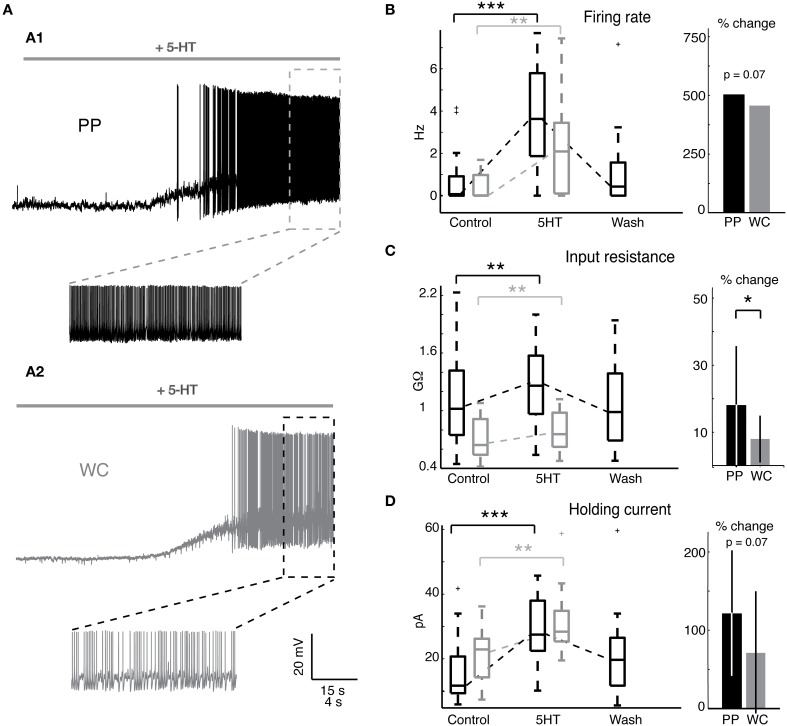
**Serotonin changes intrinsic properties in both PPR and WCR V2a INs. (A)** Sample traces from a PPR **(A1)** and a WCR **(A2)**, showing depolarization and firing in response to 10 μM 5-HT. **(B)** Serotonin increased the mean firing rate of both PP and WC cells, but the amount of change was not different between the two. The box plot shows the values of all excited cells in control conditions and in 5-HT, and the bar graph shows the mean percent change in response to 5-HT. (No error bars are included for the firing rate, as the percent increase for individual cells cannot be calculated for those cells starting with a firing rate of zero.) **(C)** Serotonin increased the input resistance of both PP and WC neurons. The increase in PP cells was significantly larger than the change in WC cells. (**D**) Both PP and WC excited cells depolarized in response to 5-HT, and therefore required an increased holding current to hyperpolarize them to −60 mV. The increase in holding current was significant for each, but the amount of change was not significantly different between the two.

We set out to determine whether PPR, which preserves the neuron's cytoplasm, would unveil a larger or qualitatively different response to 5-HT than WCR, which rapidly dialyzes out the cytoplasm within a few minutes. Serotonin (5-HT) has been previously shown to excite most V2a INs (Zhong et al., 2010). We first measured the effect of 5-HT on the intrinsic firing properties of V2a INs (Figure 3A). The majority of both PP and WC neurons responded to 5-HT with depolarization (19/20 PP neurons versus 13/16 WC neurons, Fisher's exact test *p* = 0.30). 5-HT washout typically took about one hour, but a few cells never showed complete washout, even after several hours. The remainder of the discussion of 5-HT responses refers only to the group of neurons that were excited by 5-HT.

Both PP and WC excited neurons increased their mean firing rate in response to 5-HT (Figure 3B). All excited neurons in both recording conditions, including cells that were silent in control conditions, increased their mean firing rate (Figure 3B, Wilcoxon, PPR: *p* < 0.0001; WC: *p* = 0.001). The amount of increase in the mean firing rate showed a trend to be larger in PPR, though this was not quite statistically significant (regression, *p* = 0.069). Only the PP neurons could routinely be held long enough to show a reversal of this effect.

Serotonin increased the input resistance in both PP and WC excited neurons (Figure 3C, Wilcoxon, PPR: *p* = 0.002; WCR: *p* = 0.003). In this case, the input resistance increase in PP neurons was significantly larger than the increase in WC cells (Figure 3C, regression, *p* = 0.049). Again, only the PP neurons could routinely be held long enough to show a reversal of this effect (Figure 3C).

The depolarizing response to 5HT required an increased holding current to hyperpolarize the membrane potential to −60 mV for both PP and WC neurons (Figure 3D, Wilcoxon, PPR: *p* < 0.0001; WCR: *p* = 0.0002); the amount of change showed a trend to be larger in PPR, but did not reach statistical significance (regression, *p* = 0.068).

**Figure 4:**
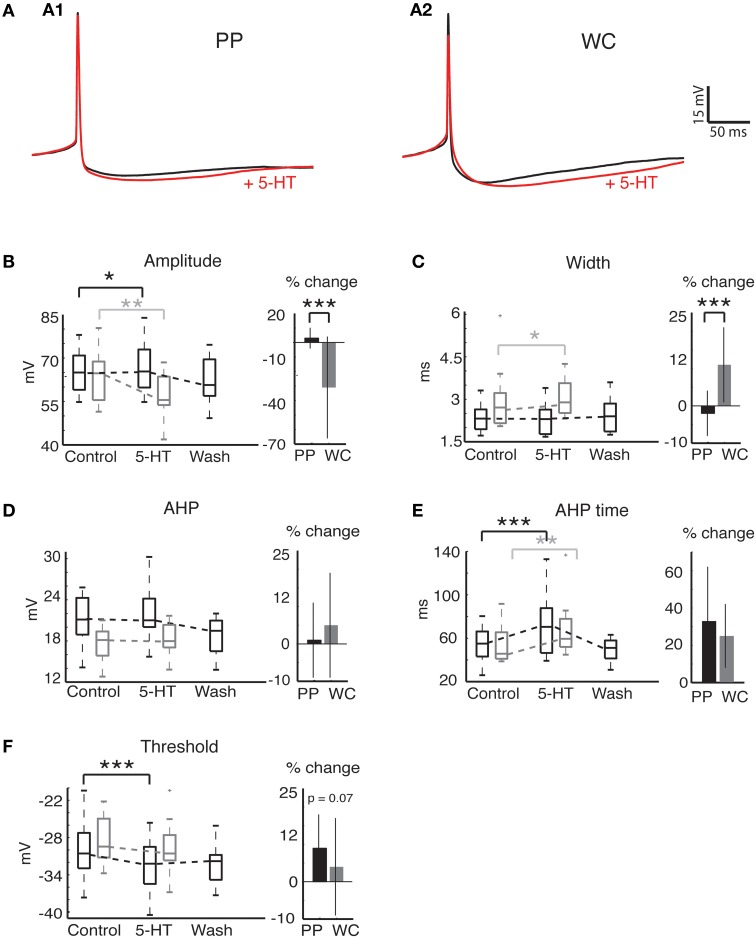
**Action potential properties change in response to 5-HT in both PP and WC V2a INs. (A)** Effects of serotonin on APs recorded in PPR **(A1)** and WCR **(A2)**. Traces are averages of 10 APs, with the average AP during 5-HT shown in red; the PPR APs are evoked at a more hyperpolarized threshold, and have been slightly depolarized to overlap the WCR APs. **(B)** The AP amplitude increased significantly with PPR and decreased significantly with WCR. The amount of change was significantly different for PPR versus WCR. **(C)** The AP width did not change significantly in PPR, but became significantly larger in WCR. The amount of change was significantly different for PPR versus WCR. **(D)** The AHP amplitude did not change significantly with either recording method. **(E)** The AHP time increased significantly in both recording methods, but the amount of change was not significantly different for PPR versus WCR. **(F)** The AP threshold became significantly more hyperpolarized in PPR but did not change significantly in WCR. The amount of change was not significantly different for PPR versus WCR.

AP properties were also affected by 5-HT using both PPR and WCR (Figure 4A). Two properties, AP amplitude and spike half-width, are closely related to access resistance and the quality of the recording. The mean AP amplitude rose slightly but significantly during 5-HT HTHT using PPR, perhaps reflecting the increased input resistance during 5-HT or an increase in the proportion of non-inactivated Na^+^ channels as a consequence of the hyperpolarization of the threshold (Figure 4B, Wilcoxon *p* = 0.049). Using WCR, the amplitude decreased significantly, and this decrease did not reverse upon wash-out of 5-HT, reflecting the decline in overall recording quality with time of WCR (Figure 4B, Wilcoxon *p* = 0.008). Similarly, AP half-width remained constant in PPR but increased slightly in WCR, presumably because of declining recording quality with time (Figure 4C, Wilcoxon *p* = 0.03). The amount of change was significantly different between PPR and WCR for both amplitude (regression *p* < 0.0001) and half-width (regression *p* < 0.0001).

Other AP properties were similarly affected by 5-HT using both PPR and WCR. The AHP amplitude did not change significantly with either recording method (Figure 4D, Wilcoxon, PPR *p* = 0.67, WCR *p* = 0.45). The time from the peak of the AP to the minimum membrane potential reached during the AHP (time) increased significantly using both recording methods, but the amount of change was not different between the two (Figure 4E, Wilcoxon, PPR *p* = 0.0003, WCR *p* = 0.0017, regression *p* = 0.66). The mean AP threshold became significantly more hyperpolarized during 5-HT using PPR (Figure 4F; Wilcoxon *p* < 0.0001). In WC neurons, the reduction in mean threshold was about half of that seen in PPR, but this reduction was not significant (Wilcoxon *p* = 0.58). Due to the large variability in response of the WC neurons, the amount of change in the threshold was not quite significantly different between PPR and WCR (regression *p* = 0.067).

Overall, we note a trend toward a larger response to 5-HT using PPR compared to WCR. Nonetheless, the magnitude of this difference was relatively small, and the responses were qualitatively similar.

### Effect of 5-HT on excitability of V2a INs using PPR and WCR

**Figure 5:**
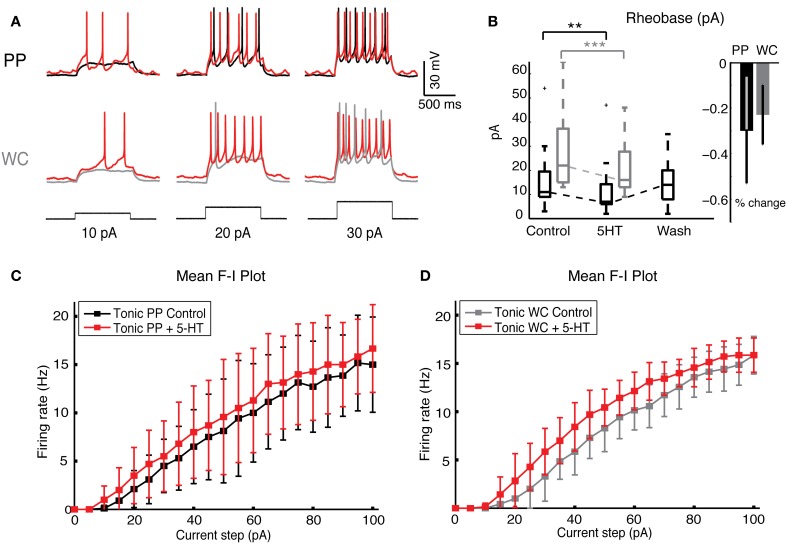
**Serotonin increases neuronal excitability in both PP and WC V2a INs. (A)** Sample traces from a PPR and from a WCR of V2a INs held at −60 mV, showing injected current steps of 10, 20, and 30 pA in black. The increase in firing during 5-HT is shown in red. **(B)** The rheobase decreased significantly with both PP and WC neurons, but the amount of change was not significantly different between the two methods. **(C,D)** The F-I plot of the mean frequency of tonic excited PP cells **(C)** and WC cells **(D)** shows a significant increase in frequency upon addition of 5-HT (instantaneous rate data not shown) (PPR: *N* = 9; WCR: *N* = 7). The extent of the increase is no different in PPR versus WCR (regression analysis, effect of recording type *p* = 0.94).

Serotonin increased the responses of PP and WC neurons to current steps by the same amount (Figure 5A). The rheobase decreased significantly during 5-HT in both PP and WC excited neurons (Figure 5B, Wilcoxon, PPR: *p* < 0.0001, WCR: *p* < 0.0001), but the amount of change was not different between the two recording methods (regression *p* = 0.28). The F-I plot of tonic V2a INs indicated increased excitability using both PPR (Figure 5C; regression, mean frequency *p* < 0.0001, instantaneous frequency *p* < 0.0001) and WCR (Figure 5D, regression, mean frequency *p* < 0.0001, instantaneous frequency *p* < 0.0001), but the extent of the increase in excitability was not different between them (regression, mean frequency *p* = 0.94, instantaneous frequency *p* = 0.85).

## Discussion

### Increased stability but similar baseline properties in PPR compared to WCR

We have compared the effects of the perforated patch recording (PPR) and WCR methods on the baseline properties and serotonin responsiveness of mouse neonatal spinal V2a INs. The most striking difference between the two recording types was that PP recordings were far longer-lasting than WCR, lasting for several hours without decrement, as we previously described for adult mouse spinal INs (Husch et al., 2011). Most of the intrinsic properties were the same using the two recording methods under control conditions (Figure 1 and Table 1). One difference between PPR and WCR was the higher input resistance of PP cells, which is expected from our previous PP studies (Husch et al., 2011) (Table 1). An unexpected difference was that the maximal AHP was more hyperpolarized using PPR (Table 1). In other cell types, the AHP is dependent on calcium-activated potassium currents and is subject to modulation via second messenger systems (Sah, 1996). We have previously observed that 5-HT indirectly reduces the amplitude of the apamin-sensitive AHP in commissural INs in neonatal mouse spinal cord (Zhong et al., 2006) via a reduction in calcium currents (Diaz-Rios et al., 2007; Abbinanti and Harris-Warrick, 2012). The difference in this AHP amplitude with the different recording methods in V2a INs may reflect a significant loss of second messenger function in WCR compared to PPR. Although all PPR cells combined showed a lower rheobase (Figure 2B), perhaps reflecting their increased input resistance due to differences in leak conductances, in the subset of V2a INs that fired tonically with all depolarizing current steps there was no difference in excitability between PPR and WCR methods (Figure 2C).

### PPR reveals subtle 5-HT dependent changes that are not visible in WCR

The majority of V2a INs recorded with both PPR and WCR were excited by 5-HT; most cells depolarized and increased their firing frequency. Among excited cells, there was a trend toward a greater 5-HT response with PPR, as seen by the significantly larger increase in input resistance (Figure 3C) and nearly significant larger increase in firing rate (Figure 3B) and holding current (Figure 3D), and hyperpolarization of threshold (Figure 4F), in PP cells. An increased response in PPR could reflect the greater efficiency of the intact intracellular machinery in transducing the 5-HT signal, or may simply be a function of the higher quality of PP recordings, even a few minutes into the recording. However, the 5-HT-induced increase in excitability during depolarizing current steps was not significantly different by recording type (Figure 5).

In the few previous studies comparing cellular responses to 5-HT in PPR and WCR, some cell types have shown increased responses to 5-HT in PPR while others showed no difference. A study of rat mesenteric artery smooth muscle cells concluded that retaining the cell's native calcium buffering in PPR yielded an enhanced 5-HT-induced depolarization over WCR (Bae et al., 2006). In voltage clamp studies of ventromedial hypothalamic neurons, 5-HT inhibited both N- and P/Q-type HVA-Ca currents when measured by PPR, whereas in previous studies using WCR, only N-type currents were inhibited by 5-HT (Koike et al., 1994; Rhee et al., 1996); this difference appears to reflect the loss of a modulatory effect of 5-HT after dialysis of the cell cytoplasm. However, in fast-spiking GABAergic INs in rat striatum, the amplitude and time course of 5-HT-induced depolarization in the eight cells recorded in PPR were not different from those recorded in WCR (Blomeley and Bracci, 2009).

### The 5-HT receptor subtypes and currents involved

The full complement of 5-HT receptor subtypes expressed in V2a INs, and the currents that they modulate, are not yet known. In the best-characterized neuron type in the spinal cord, motoneurons, 5-HT mediates excitability via its effects on an array of currents, including persistent calcium currents (Li et al., 2007), persistent sodium currents (Harvey et al., 2006), and the closure of potassium leak channels (VanderMaelen and Aghajanian, 1980; Perrier et al., 2003). In mouse commissural INs, 5-HT causes excitation by a reduction in calcium-activated potassium currents (Diaz-Rios et al., 2007) as a consequence of reduction of N- and P/Q-type calcium currents (Abbinanti and Harris-Warrick, 2012). Potassium current reduction is particularly promising as a contributor to V2a excitation because it would account for the increase in input resistance during application of 5-HT.

## Conclusion

In theory, PPR should provide a more faithful representation of the effects of neuromodulators on the firing properties of neurons, since the neuronal cytoplasm is essentially unaffected by the recording, whereas WCRs rapidly dialyze the cytoplasm from the cell soma. In practice, we found that the V2a neurons retained their normal firing properties for much longer during PPR than WCR. While there was a trend toward stronger V2a responses to 5-HT in PPR recordings, the absolute differences between PPR and WCR responses were quantitative rather than qualititative. We conclude that WCR is adequate for measuring 5-HT responses over short recording times, when 5-HT washout is not necessary. However, for the maximum 5-HT response, and for any experiment in which high-quality recordings are necessary over the time course of hours, allowing for wash on and washout of any applied drugs, PPR is crucial for success.

### Conflict of interest statement

The authors declare that the research was conducted in the absence of any commercial or financial relationships that could be construed as a potential conflict of interest.

## References

[B1] Abbinanti M. D. Harris-Warrick R. M. (2012). Serotonin modulates multiple calcium current subtypes in commissural interneurons of the neonatal mouse. J. Neurophysiol. 107, 2212–2219 10.1152/jn.00768.2011 22279189 PMC3331599

[B2] Al-Mosawie A. Wilson J. M. Brownstone R. M. (2007). Heterogeneity of V2-derived interneurons in the adult mouse spinal cord. Eur. J. Neurosci. 26, 3003–3015 10.1111/j.1460-9568.2007.05907.x 18028108

[B3] Aosaki T. Kiuchi K. Kawaguchi Y. (1998). Dopamine D1-like receptor activation excites rat striatal large aspiny neurons *in vitro*. J. Neurosci. 18, 5180–5190 9651201 10.1523/JNEUROSCI.18-14-05180.1998 PMC6793488

[B4] Armstrong D. Eckert R. (1987). Voltage-activated calcium channels that must be phosphorylated to respond to membrane depolarization. Proc. Natl. Acad. Sci. U.S.A. 84, 2518–2522 2436233 10.1073/pnas.84.8.2518 PMC304685

[B5] Bae Y. M. Kim A. Kim J. Park S. W. Kim T. K. Lee Y. R. (2006). Serotonin depolarizes the membrane potential in rat mesenteric artery myocytes by decreasing voltage-gated K+ currents. Biochem. Biophys. Res. Commun. 347, 468–476 10.1016/j.bbrc.2006.06.116 16828462

[B6] Becq F. (1996). Ionic channel rundown in excised membrane patches. Biochim. Biophys. Acta 1286, 53–63 8634323 10.1016/0304-4157(96)00002-0

[B7] Belles B. Hescheler J. Trautwein W. Blomgren K. Karlsson J. O. (1988a). A possible physiological role of the Ca-dependent protease calpain and its inhibitor calpastatin on the Ca current in guinea pig myocytes. Pflugers Arch. 412, 554–556 2848213 10.1007/BF00582548

[B8] Belles B. Malecot C. O. Hescheler J. Trautwein W. (1988b). “Run-down” of the Ca current during long whole-cell recordings in guinea pig heart cells: role of phosphorylation and intracellular calcium. Pflugers Arch. 411, 353–360 2456513 10.1007/BF00587713

[B9] Blomeley C. P. Bracci E. (2009). Serotonin excites fast-spiking interneurons in the striatum. Eur. J. Neurosci. 29, 1604–1614 10.1111/j.1460-9568.2009.06725.x 19419423 PMC2695856

[B10] Chad J. Kalman D. Armstrong D. (1987). The role of cyclic AMP-dependent phosphorylation in the maintenance and modulation of voltage-activated calcium channels. Soc. Gen. Physiol. Ser. 42, 167–186 2850609

[B11] Chad J. E. Eckert R. (1986). An enzymatic mechanism for calcium current inactivation in dialysed Helix neurones. J. Physiol. 378, 31–51 2432251 10.1113/jphysiol.1986.sp016206 PMC1182851

[B12] Crone S. A. Quinlan K. A. Zagoraiou L. Droho S. Restrepo C. E. Lundfald L. (2008). Genetic ablation of V2a ipsilateral interneurons disrupts left-right locomotor coordination in mammalian spinal cord. Neuron 60, 70–83 10.1016/j.neuron.2008.08.009 18940589

[B13] Crone S. A. Zhong G. Harris-Warrick R. Sharma K. (2009). In mice lacking V2a interneurons, gait depends on speed of locomotion. J. Neurosci. 29, 7098–7109 10.1523/JNEUROSCI.1206-09.2009 19474336 PMC2731420

[B14] Diaz-Rios M. Dombeck D. A. Webb W. W. Harris-Warrick R. M. (2007). Serotonin modulates dendritic calcium influx in commissural interneurons in the mouse spinal locomotor network. J. Neurophysiol. 98, 2157–2167 10.1152/jn.00430.2007 17581844

[B15] Ding L. Perkel D. J. (2002). Dopamine modulates excitability of spiny neurons in the avian basal ganglia. J. Neurosci. 22, 5210–5218 10.1016/j.brainres.2007.04.025 12077216 PMC6757730

[B16] Goulding M. (2009). Circuits controlling vertebrate locomotion: moving in a new direction. Nat. Rev. Neurosci. 10, 507–518 10.1038/nrn2608 19543221 PMC2847453

[B17] Gulledge A. T. Jaffe D. B. (1998). Dopamine decreases the excitability of layer V pyramidal cells in the rat prefrontal cortex. J. Neurosci. 18, 9139–9151 9787016 10.1523/JNEUROSCI.18-21-09139.1998 PMC6793538

[B18] Hamill O. P. Marty A. Neher E. Sakmann B. Sigworth F. J. (1981). Improved patch-clamp techniques for high-resolution current recording from cells and cell-free membrane patches. Pflugers Arch. 391, 85–100 6270629 10.1007/BF00656997

[B19] Harvey P. J. Li X. Li Y. Bennett D. J. (2006). 5-HT2 receptor activation facilitates a persistent sodium current and repetitive firing in spinal motoneurons of rats with and without chronic spinal cord injury. J. Neurophysiol. 96, 1158–1170 10.1152/jn.01088.2005 16707714 PMC5726401

[B20] Hochman S. (2001). 5-HT receptors and the neuromodulatory control of spinal cord function, in Motor Neurobiology of the Spinal Cord, ed Cope T. C. (Boca Raton, FL: CRC Press), 47–87

[B21] Horn R. Marty A. (1988). Muscarinic activation of ionic currents measured by a new whole-cell recording method. J. Gen. Physiol. 92, 145–159 2459299 10.1085/jgp.92.2.145 PMC2228899

[B22] Husch A. Cramer N. Harris-Warrick R. M. (2011). Long-duration perforated patch recordings from spinal interneurons of adult mice. J. Neurophysiol. 106, 2783–2789 10.1152/jn.00673.2011 21900514 PMC3214093

[B23] Ingram S. L. Prasad B. M. Amara S. G. (2002). Dopamine transporter-mediated conductances increase excitability of midbrain dopamine neurons. Nat. Neurosci. 5, 971–978 10.1038/nn920 12352983

[B24] Jacobs B. L. Fornal C. A. (1993). 5-HT and motor control: a hypothesis. Trends Neurosci. 16, 346–352 7694403 10.1016/0166-2236(93)90090-9

[B25] Jiang Z. Rempel J. Li J. Sawchuk M. A. Carlin K. P. Brownstone R. M. (1999). Development of L-type calcium channels and a nifedipine-sensitive motor activity in the postnatal mouse spinal cord. Eur. J. Neurosci. 11, 3481–3487 10.1046/j.1460-9568.1999.00765.x 10564356

[B26] Kiehn O. (2006). Locomotor circuits in the mammalian spinal cord. Annu. Rev. Neurosci. 29, 279–306 10.1146/annurev.neuro.29.051605.112910 16776587

[B27] Koike H. Saito H. Matsuki N. (1994). 5-HT1A receptor-mediated inhibition of N-type calcium current in acutely isolated ventromedial hypothalamic neuronal cells. Neurosci. Res. 19, 161–166 8008244 10.1016/0168-0102(94)90139-2

[B28] Korn S. J. Horn R. (1989). Influence of sodium-calcium exchange on calcium current rundown and the duration of calcium-dependent chloride currents in pituitary cells, studied with whole cell and perforated patch recording. J. Gen. Physiol. 94, 789–812 2556494 10.1085/jgp.94.5.789 PMC2228975

[B29] Korn S. J. Weight F. F. (1987). Patch-clamp study of the calcium-dependent chloride current in AtT-20 pituitary cells. J. Neurophysiol. 58, 1431–1451 2449518 10.1152/jn.1987.58.6.1431

[B30] Kudo N. Yamada T. (1987). N-methyl-D, L-aspartate-induced locomotor activity in a spinal cord-hindlimb muscles preparation of the newborn rat studied *in vitro*. Neurosci. Lett. 75, 43–48 3554010 10.1016/0304-3940(87)90072-3

[B31] Li X. Murray K. Harvey P. J. Ballou E. W. Bennett D. J. (2007). Serotonin facilitates a persistent calcium current in motoneurons of rats with and without chronic spinal cord injury. J. Neurophysiol. 97, 1236–1246 10.1152/jn.00995.2006 17079337 PMC5718189

[B32] Lindau M. Fernandez J. M. (1986). IgE-mediated degranulation of mast cells does not require opening of ion channels. Nature 319, 150–153 10.1038/319150a0 2417125

[B33] Liu Y. Lasater E. M. (1994). Calcium currents in turtle retinal ganglion cells. II. Dopamine modulation via a cyclic AMP-dependent mechanism. J. Neurophysiol. 71, 743–752 8176436 10.1152/jn.1994.71.2.743

[B34] Lucero M. T. Pappone P. A. (1989). Voltage-gated potassium channels in brown fat cells. J. Gen. Physiol. 93, 451–472 2467964 10.1085/jgp.93.3.451 PMC2216218

[B35] Lucero M. T. Pappone P. A. (1990). Membrane responses to norepinephrine in cultured brown fat cells. J. Gen. Physiol. 95, 523–544 1969922 10.1085/jgp.95.3.523 PMC2216326

[B36] Lundfald L. Restrepo C. E. Butt S. J. Peng C. Y. Droho S. Endo T. (2007). Phenotype of V2-derived interneurons and their relationship to the axon guidance molecule EphA4 in the developing mouse spinal cord. Eur. J. Neurosci. 26, 2989–3002 10.1111/j.1460-9568.2007.05906.x 18028107

[B37] Moyer J. R. Brown T. (2002). Patch-clamp techniques applied to brain slices, in Patch-Clamp Analysis: Advanced Techniques, eds Boulton A. A. Baker G. B. Walz W. (Totowa, NJ: Humana Press), 135–193

[B38] Neter J. Kutner M. H. Nachtsheim C. J. Wasserman W. (1996). Applied Linear Regression Models. New York, NY: McGraw-Hill

[B39] Perrier J. F. Alaburda A. Hounsgaard J. (2003). 5-HT1A receptors increase excitability of spinal motoneurons by inhibiting a TASK-1-like K+ current in the adult turtle. J. Physiol. 548, 485–492 10.1113/jphysiol.2002.037952 12626670 PMC2342869

[B40] Rae J. Cooper K. Gates P. Watsky M. (1991). Low access resistance perforated patch recordings using amphotericin B. J. Neurosci. Methods 37, 15–26 10.1016/0165-0270(91)90017-T 2072734

[B41] Rhee J. S. Ishibashi H. Akaike N. (1996). Serotonin modulates high-voltage-activated Ca2+ channels in rat ventromedial hypothalamic neurons. Neuropharmacology 35, 1093–1100 10.1016/S0028-3908(96)00052-4 9121612

[B42] Sah P. (1996). Ca(2+)-activated K+ currents in neurones: types, physiological roles and modulation. Trends Neurosci. 19, 150–154 10.1016/S0166-2236(96)80026-9 8658599

[B43] Sarantopoulos C. (2007). Perforated patch-clamp techniques, in Patch-Clamp Analysis: Advanced Techniques, ed Walz W. (Totowa, NJ: Humana Press), 253–293

[B44] Schmidt B. J. Jordan L. M. (2000). The role of serotonin in reflex modulation and locomotor rhythm production in the mammalian spinal cord. Brain Res. Bull. 53, 689–710 10.1016/S0361-9230(00)00402-0 11165804

[B45] VanderMaelen C. P. Aghajanian G. K. (1980). Intracellular studies showing modulation of facial motoneurone excitability by serotonin. Nature 287, 346–347 7421993 10.1038/287346a0

[B46] Zhong G. Diaz-Rios M. Harris-Warrick R. M. (2006). Serotonin modulates the properties of ascending commissural interneurons in the neonatal mouse spinal cord. J. Neurophysiol. 95, 1545–1555 10.1152/jn.01103.2005 16338993

[B47] Zhong G. Droho S. Crone S. A. Dietz S. Kwan A. C. Webb W. W. (2010). Electrophysiological characterization of V2a interneurons and their locomotor-related activity in the neonatal mouse spinal cord. J. Neurosci. 30, 170–182 10.1523/JNEUROSCI.4849-09.2010 20053899 PMC2824326

